# Clinical Analysis of Minimally Invasive Percutaneous Treatment of Severe Lumbar Disc Herniation with UBE Two-Channel Endoscopy and Foraminal Single-Channel Endoscopy Technique

**DOI:** 10.1155/2022/9264852

**Published:** 2022-10-13

**Authors:** Cuihua Yuan, Baojun Wen, Hongkuan Lin

**Affiliations:** Department of Orthopaedics, Mindong Hospital Affiliated to Fujian Medical University, Fuan, 355000 Fujian, China

## Abstract

For surgical treatment, herniation of traditional open surgery is the traditional approach and the representative operation for lamina windowing excision of nucleus pulposus. In recent years, the unilateral dual channel spine endoscopic technology (UBE/BESS) has caused extensive concern of spine surgery performer. This research compared the results of minimally invasive percutaneous treatment of severe lumbar disc herniation with foraminal single-channel endoscopy and unilateral biportal endoscopy (UBE). A retrospective study was conducted on 50 patients with severe disc herniation treated with minimally invasive percutaneous treatment in MinDong Hospital affiliated to Fujian Medical University from September 2019 to September 2021. According to different surgical methods, they were divided into two groups: foraminal single-channel endoscopic group and UBE dual-channel endoscopic group. There were 22 cases in the UBE surgery group and 28 cases in the interforaminal endoscopic group. The comparison included operation time, postoperative hospital stays, preoperative and postoperative pain scale (VAS), and postoperative MRI to observe the residual condition of prolapsed nucleus pulposus and the occurrence of complications. There were no significant differences between the UBE group and the interforaminal endoscopic group in incision length, operation time, postoperative hospital stays, and improvement of VAS score before and after surgery. In terms of postoperative nucleus pulposus residual rate and postoperative recurrence rate, the two-channel UBE group was significantly better than the single-channel interforaminal endoscopic group. The incidence of postoperative anemia in the interforaminal endoscopic group was significantly lower than that in the UBE group. In the treatment of severe disc herniation, UBE two-channel endoscopy has the advantages of lower recurrence rate, lower nucleus pulposus residual rate, shorter learning curve, and better field of vision than foraminal single-channel endoscopy, which is worth promoting in primary hospitals.

## 1. Introduction

Lumbar disc herniation (LDH), a local displacement of intervertebral disc material beyond the normal intervertebral disc space edge, usually causes low back pain and radiculopathy [1]. It is the most common cause of sciatica, affecting 1% to 5% of the population every year [2]. Lumbar disc herniation often dissolves over time, and the spontaneous resorption rate is 60% or above [3]. The first-line treatment for sciatica is non-surgical treatment, including physical therapy, drug therapy, and epidural steroid injection [4, 5], whereas surgery can relieve symptoms faster than continuous conservative treatment [6]. However, after a long follow-up, the differences between the groups tended to converge, but they still preferred surgical treatment.

For surgical treatment, herniation of traditional open surgery is the traditional approach and the representative operation for lamina windowing excision of nucleus pulposus. In traditional open surgery, there are many possible complications, such as excessive loss of intraoperative vertebral side, long-time muscle exertion, cerebrospinal fluid leakage, vertebral instability, low back pain and recurrence [7, 8]. Spinal endoscopy is a new minimally invasive spinal technology. Yeung [9] first proposed the Yeung endoscopy spine system (YESS) technology through the kambin triangle safety zone [10] in 1998. In 2002, Hoogl-and et al. proposed the technique of Transforaminal Endoscopic Spine System (TESSYS) [11, 12].

In recent years, the Unilateral Biportal Endoscopic (UBE/BESS) technology has caused extensive concern of spine surgery performer. UBE is unilateral dual-channel endoscopic technology (unlateral biportal endoscopic technique); the technology is usually set up two channels, a channel for observation and an instrument operating channels. Observation channels generally use 0° or 30° of arthroscopy, now can be very good completed the mirror through UBE technology fusion surgery under [13, 14].

At present, there are few comparative reports in the literature on the efficacy of UBE two-channel endoscopy and foraminal single-channel endoscopy technique in the treatment of severe intervertebral disc herniation. Thus, the motivation and novelty of this paper is to compare the efficacy of the above two minimally invasive endoscopic techniques in the treatment of severe intervertebral disc herniation.

## 2. Materials and Methods

### 2.1. General Information

The spine surgery retrospective comparative analysis of MinDong Hospital between September 2019 and September 2021 of patients with severe disc herniation percutaneous minimally invasive treatment included a total of 50 cases. The ratio of male to female is 4 : 6. The age was 28–66 years old, with an average age of 38.6 years: responsible intervertebral space distribution: 8 cases of L3/4 space; L4/5 space 22 cases; L5/S1 space in 20 cases. According to the operation plan, 28 cases underwent nucleus pulposus extraction assisted by foraminal single-channel endoscopy, and 22 cases underwent UBE two-channel endoscopy-assisted nucleus pulposus extraction. There was no significant difference in gender, age, BMI, and prominent responsibility gap between the two groups. Details are shown in Table 1.

### 2.2. Case Selection

1. Case Inclusion Criteria: Our spine team operation cases showed that severe intervertebral disc hernia and hernia of nucleus pulposus arrived after the upper and lower adjacent vertebral bodies (arrived at 1 and 4 and beyond)
2. Case Exclusion Criteria: Severe osteoporosis, spinal instability, or slippage patients always received spinal open surgery. Patient age is high and accompanied by severe basic diseases, which cannot tolerate surgery

### 2.3. Case Grouping

**Figure 1:**
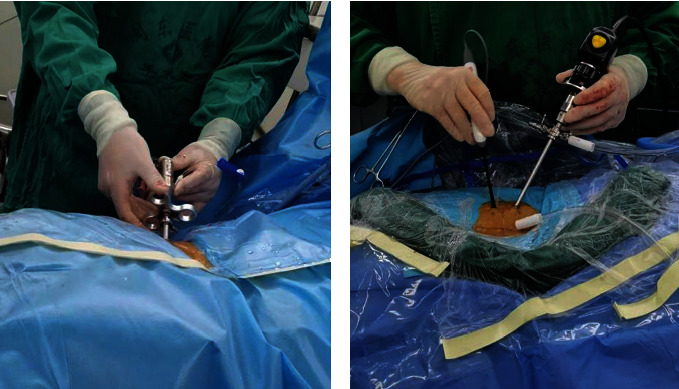
Intraoperative operation of the two approach (a: foraminal single-channel endoscopy; b: two-channel UBE.).

**Figure 2:**
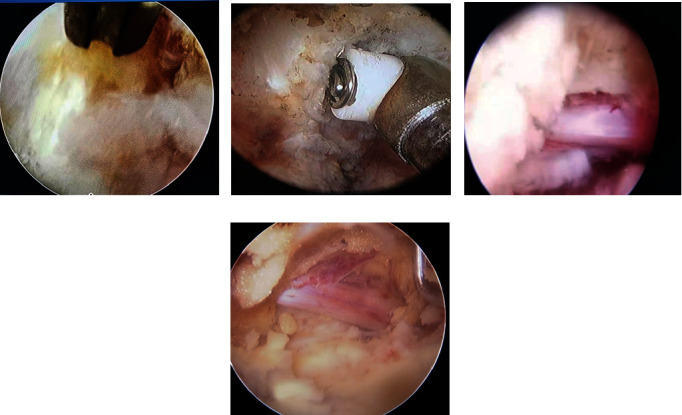
Visual field during operation (a: visual field under radiofrequency cauterization in poroscopy; b: visual field under radiofrequency ablation during UBE; c: visual field after decompression of nerve was obtained during poroscopy; d: visual field after decompression of nerve was obtained during UBE.).

1. UBE Group: UBE under arthroscopy-assisted technology, unilateral double channel endoscopic technique, percutaneous nucleus pulposus enucleation, preoperative signed informed consent UBE technology, preoperative examination lumbar positive side, a song, ghost piece of lumbar vertebra CT, preoperative check lumbar MR know I of nucleus pulposus, and postoperative lumbar MRI review about removal of nucleus pulposus and residue are included in this group.
2. Interforaminal Endoscopic Group: Percutaneous enucleation of nucleus pulposus was performed by spinal foraminal endoscope-assisted single-channel endoscopy. Preoperative signed informed consent intervertebral foramen lens technology, preoperative signed informed consent UBE technology, preoperative examination lumbar positive side, a ghost piece, lumbar CT, lumbar MRI, and postoperative lumbar MRI review about removal of nucleus pulposus and residue are included in this group. Intraoperative operation and visual field are shown in Figures 1 and 2

### 2.4. Method

#### 2.4.1. Interforaminal Endoscopic Group (Foraminal Single-Channel Endoscopy Technology Group)

In L3 and L4/4/5 clearance, 12 cases were treated with topical anesthesia in 5 cases with local anesthesia plus intravenous reinforcement; 11 cases adopted general anesthesia. All patients took the prone position, and L5/S1 clearance do vertebral plate gap into the way, according to the conventional uniaxial intervertebral foramen mirror assisted surgery and according to the observation of intraoperative nerve root beat situation to judge the nerve root decompression are in good condition.

#### 2.4.2. UBE Group (UBE Two-Channel Technology Group)

All cases underwent endotracheal intubation anesthesia and surgery in the prone position. Two skin incisions were made under C-arm fluoroscopic guidance. The initial target point of the mirror instrument is located at the junction of the spinous process and the lamina, so as to make a horizontal marking line and draw a marking line along the inner edge of the pedicle. Intersection of two lines and 1.5 cm, respectively, to observe incision and incision operation point of the body surface. And we made 2 portals, layer by layer, the lumbar and dorsal fascia was cut longitudinally, and the soft tissue covered by the bony surface of the lamina was gradually expanded and bluntly separated to form the observation portals and the working portals. In the observation portals, the arthroscopic system was inserted and use salt water irrigation, under the hydraulic pressure to make tiny intra-spinal canal vein, does not ooze blood; it keeps to the field of vision clear; smooth flow of water is the key to UBE to get clear operative field. In the working portals, the soft tissue on the surface of the intervertebral space was treated under the 90° plasma scalpel and hemostasis was performed. Lamina rongeur and arthroscopy of the operating system dynamic power drill were used to remove the target intervertebral disc under the upper edge of vertebral lamina and a vertebral lamina edge, the edge of exposure on the yellow ligament, and the removal of yellow ligament. Using nerve hook open, nerve root, and dural sac intervertebral disc, preoperative MRI image data suggest peering into spinal canal and vertebral bodies with nucleus pulposus clamp fall off removal of nucleus pulposus, under endoscopic direct from removed off to the nucleus pulposus in the back part of the vertebral bodies.

**Figure 3:**
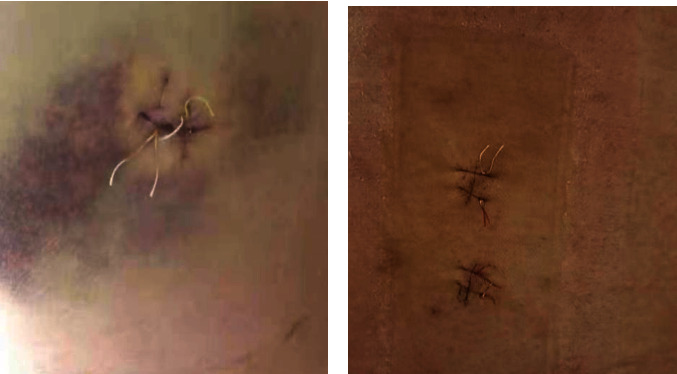
Incision of two approach (a: incision after single-channel endoscopy; b: incision after two-channel UBE.).

**Figure 4:**
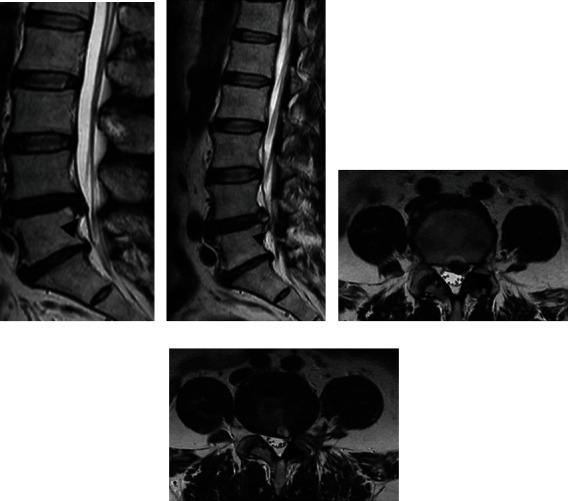
MRI imaging of typical case 1 (a: MRI sagittal of L4. Five intervertebral disc herniation before operation; b: MRI sagittal of L4. Five intervertebral disc after prolapse poroscopy; c: coronal MRI of L4. Five intervertebral disc herniation before operation; d: coronal MRI of L4. Five intervertebral disc herniation after prolapse poroscopy.).

Typical Case 1: Patient, male, 46 years old, L4 5. After intervertebral disc prolapse and intervertebral foraminal endoscopic nucleus pulposus extraction (Figures 3 and 4), MRI lost its shape and position before and after operation. It can be seen that there are still some residual compression nerve roots of nucleus pulposus after operation.

**Figure 5:**
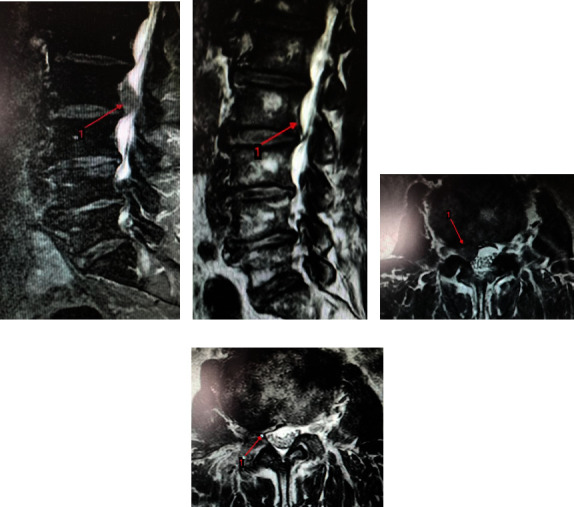
MRI imaging of typical case 1 (a: MRI sagittal of L2. Three intervertebral disc herniation before operation; b: MRI sagittal of L2. Three intervertebral disc after prolapse poroscopy; C: coronal MRI of L2. Three intervertebral disc herniation before operation; D: coronal MRI of L2. Three intervertebral disc herniation after prolapse poroscopy.).

Typical case 2: Patients, male, 60 L2.3 slipped disc, line UBE dual channel after percutaneous minimally invasive nucleus pulposus enucleation (Figure 5), preoperative and postoperative MRI 3 loss with coronary, visible after removal of nucleus pulposus thoroughly, and no leave of nucleus pulposus.

### 2.5. Observation Index

1. Effect of Evaluation Method: namely, operation time, intraoperative blood loss (loss with Hgb hemoglobin before and after operation situation assessment), incision length, operation rate, and hospital stay after operation
2. Postoperative Review: magnetic resonance imaging (MRI) watch out residue and complications of nucleus pulposus; cerebrospinal fluid leakage after operation was compared between the two groups
3. Follow-Up Records: VAS score (visual analogue pain score) was recorded before operation, after discharge, and 3 months and 6 months after operation. On a scale of 10, 0 indicates no pain and 10 indicates severe pain, with the end of the month being less painful

### 2.6. Statistical Analysis

The SPSS 16.0 statistical software was used for data analysis, and the measurement data were expressed in *x* ± *s*. Rank sum test or *t*-test was used for measurement data, and intergroup comparison and counting data used *χ*^2^ inspection. When the statistical result is *P* < 0.05, it is considered that the difference is statistically significant.

## 3. Results

### 3.1. Clinical Information

Two groups of operation time, postoperative hospitalization days, preoperative and postoperative VAS score difference have no obvious statistical significance (*P* > 0.05). Two groups of postoperative incision length difference were statistically significant (*P* < 0.05) (see Table 2).

### 3.2. Intraoperative Operation and Postoperative Complications

The UBE group under the dual-channel operation, wide field of vision, and flexible operating instrument freedom is not restricted. The UBE group of cerebrospinal fluid leakage in the postoperative complications occurred in 2 cases, The interforaminal endoscopic group was 0 cases; the occurrence of infection in the two groups was 0 cases; nucleus pulposus residues in the UBE group occurred in 0 cases; the interforaminal endoscopic group occurred in 7 cases. The complication rate of two groups showed no significant difference (*P* = 0.384); however, there were significant differences in the incidence of anemia and residual nucleus pulposus between the two groups (as shown in Table 3).

## 4. Discussion

### 4.1. Advantages and Disadvantages of UBE Compared with Interforaminal Endoscopic

Compared with UBE two-channel endoscopic technique and foraminal single-channel endoscopy technique, the two techniques have the following advantages and disadvantages: The advantages of percutaneous minimally invasive technique with uniaxial foraminal endoscope in the treatment of spinal diseases are minimally invasive, less intraoperative bleeding, and no need to place drainage ball after operation. The probability of postoperative anemia is low. The operation can be completed under local anesthesia.

Compared with UBE technology, the shortcomings of uniaxial foraminal endoscopic percutaneous minimally invasive technology are as follows: Uniaxial intervertebral foraminal mirror equipment is relatively expensive and has a long learning curve. Most grass-roots county-level hospitals have not purchased the equipment and still cannot master the technology in most hospitals. In addition, the field of vision of single-channel intervertebral foraminal endoscopy is relatively limited, and the use of posterior approach is generally limited to L5S1 segment. In the segment above L5S1, posterior surgery cannot be performed because the posterior lamina space is relatively narrow. If posterior surgery is required, a special large channel (delta channel) needs to be used. Therefore, the lateral intervertebral foramen approach is generally used in clinic. When the severe intervertebral disc prolapses to the back of the vertebral body (when the nucleus pulposus falls off to zone 1 and zone 4), it is often very difficult to completely remove the prolapsed nucleus pulposus by using the lateral intervertebral foramen mirror, which requires superb endoscopic technology. Generally, it is based on the experience of the operator and the observation of the pulsation of the nerve root during the operation to judge whether the nucleus pulposus is completely removed, and there is a high possibility of residual nucleus pulposus. High iliac crest should be considered in lateral foraminal endoscopy. If there is high iliac crest obstruction, it is difficult to place the tube at the target point of lateral approach and operate under the microscope.

The advantages of UBE two-channel endoscopy compared with the interforaminal endoscopic technology are as follows: First, because the two-channel spinal endoscopy technology uses the arthroscopic operating system, the equipment is cheap, and the equipment is available in most grass-roots county hospitals. Second, UBE percutaneous minimally invasive technology itself is a microscopic surgical posterior spinal operation technology under the guidance of water medium. The intraoperative anatomy is almost the same as that of the traditional posterior spinal technology. The technology can be carried out after a short training of spinal surgeons on the basis of arthroscopy. Therefore, the learning curve of UBE is relatively flat, and the learning cycle is short. Third, the essence of UBE is the surgical posterior spinal technique under the microscope. The field of vision under the double-channel microscope is more comprehensive, and the nucleus pulposus can be removed completely under direct vision. The surgical field of vision is wide, and the probability of residual nucleus pulposus is very low. Especially when the nucleus pulposus is prolapsed to the back of the vertebral body (nucleus pulposus falls off to zone 1 and zone 4) in the treatment of severe intervertebral disc herniation, UBE has obvious advantages, Tian, Zhu, and others also proposed that UBE has a good effect in the treatment of prolapsed free disc herniation [15, 16]. Fourth, UBE has many advantages, such as more flexible operation and more choice of instruments, which has more advantages in the treatment of lumbar spinal stenosis. In the future, it can enable more and more complex spinal degenerative diseases to achieve minimally invasive and endoscopic treatment. Fifth, the posterior approach of UBE operation does not need to consider the blocking factor of high iliac crest.

UBE has the following disadvantages compared with intervertebral foramen technology: First, the trauma of UBE is relatively larger than that of poroscopy: two incisions should be made during the operation. During the operation, the muscle attached to the lamina needs to be stripped under the microscope, and the artificial creation of two operation spaces needs to damage and destroy the anatomical structure of multifidus muscle and longissimus pectoralis muscle. Part of the vertebral lamina needs to be chiseled during the operation, and there is iatrogenic injury. There is relatively more bleeding during the operation than the hole mirror technology. After the operation, it is often necessary to place a drainage ball to prevent intraspinal hematoma. Second, UBE technology is relatively traumatic, and the probability of postoperative anemia is higher than that of poroscopy. Third, UBE technology generally needs to be carried out under general anesthesia, and lateral intervertebral foramen technology can be carried out under local anesthesia. UBE posterior surgery is a microscopic posterior spinal surgery. During the operation, it is often necessary to chisel out the lamina and remove the ligamentum flavum. The probability of cerebrospinal fluid leakage caused by dural injury is relatively high.

### 4.2. Progress of UBE

De Antoni et al. [17] first reported UBE in 1996 and achieved good curative effect. In 1996, they performed discectomy under dual channel arthroscopy and achieved good clinical effect. They proposed dual channel technology to improve the vision and flexibility of operation.

Soliman first proposed the application of this technology to the minimally invasive treatment of intervertebral disc herniation in 2013 [18]. He proposed that the operation uses water as the medium, and the two-channel percutaneous minimally invasive technology expands the surgical field of vision, with less vascular bleeding in the spinal canal under the pressure of water medium. Eum et al. [19] applied the percutaneous dual-channel spinal endoscopic decompression technology to the treatment of lumbar spinal stenosis and found that under the assistance of percutaneous dual-channel endoscopic technology, the vertebral lamina can be windowed and decompressed through one incision, and the bottom of spinous process base can be bitten off through lamina gun pliers to the opposite side and bilateral decompression and ULBD can be performed without bilateral incision, and there is less vascular bleeding and less trauma under water pressure. In 2017, Heo et al. [13] first proposed the concept of unilateral dual channel endoscopy. At present, unilateral and dual-channel endoscopy is widely used in the treatment of lumbar spinal stenosis and lumbar disc herniation, with good curative effect [20, 21].

### 4.3. Surgical Techniques of UBE Single-Side Dual-Channel Spinal Endoscopy

The following points should be paid attention to in the operation of UBE percutaneous minimally invasive technique in the treatment of severe lumbar disc herniation:

1. During preoperative positioning, the target space should be perpendicular to the ground, and the two channels should be V-shaped
2. Before the operation, the C-arm machine transmission was clear and the operation gap was correct. During the operation, the operation space was created and the operation gap was clear again
3. Keeping the water flowing smoothly and maintaining the stability of water pressure is the key to obtain a clear surgical field. During the operation, it is necessary to completely cut the low back fascia with a sharp knife and make a “cross” incision on the low back fascia under the incision with a sharp knife, which can keep the water flowing smoothly. At the same time, during the operation, pay attention to remove the soft tissue near the channel outlet to avoid the soft tissue blocking the water outlet, resulting in blurred vision. Especially at the beginning of the operation, the soft tissue attached to the upper and lower edges of the vertebral lamina needs to be stripped with a plasma knife. At this time, the detached soft tissue will block the water outlet, resulting in blurred vision at the beginning of the operation, so that the north and south cannot be distinguished during the operation. At this time, the operator should be calm and completely remove the soft tissue near the water outlet channel with nucleus pulposus forceps to keep the water outlet unobstructed and the field of vision variable and clear
4. MRI and other imaging data should be carefully read before operation to clarify the location of the detached free nucleus pulposus, so that we can know well when exploring and removing the detached nucleus pulposus during operation

### 4.4. Limitations and Prospects

This study still has room for improvement. First, the sample size of the study is too small to detect a difference between both groups. Second, the follow-up time was too short to obtain long-term efficacy of the two surgical approaches. Besides, relatively few measures have been used to evaluate efficacy, and patient functional measures have not been discussed. Consequently, a well-designed, randomized, and controlled trial with prospective data collection and sample size calculation is needed to confirm the findings in our study and to examine the long-term efficacy of two approaches with clinical outcomes.

## 5. Conclusion

In the treatment of severe disc herniation, UBE unilateral two-channel percutaneous minimally invasive technology has wider field of vision, low residual rate of nucleus pulposus, and short learning curve compared with foraminal single-channel endoscopy. In the UBE group compared with the interforaminal endoscopic group, intraoperative on bone and soft tissue damage is bigger; the intraoperative bleeding is more, a high incidence of postoperative anemia; the damage of epidural cerebrospinal fluid leak rate is relatively high.

## Figures and Tables

**Table 1:** Comparison of two groups of general data.

Group	Age (year, *x* ± *s*)	Gender(male/female)	Protruding space		
			L3/4 and L2/3	L4/5	L5/S1
UBE group (*n* = 22)	40.0 ± 7.22	10/12	3	10	9
Interforaminal endoscopic group (*n* = 28)	38.0 ± 6.71	13/15	5	12	11
*t*/*χ*^2^	1.012	0.005	0.164		
*P* value	0.317	0.945	0.921		

**Table 2:** Comparison of intraoperative and postoperative conditions between the two groups ($\bar{x}\pm s$).

Group	Case	Operation time (min)	Postoperative hospital stay (d)	Preoperative/postoperative VAS score	Surgical incision (cm)
UBE group	22	65.6 ± 10.2	3.0 ± 1.5	7.02 ± 0.35/1.05 ± 0.54∗	2.26 ± 1.05
Interforaminal endoscopic group	28	62.3 ± 8.7	3.6 ± 1.2	8.13 ± 0.67/1.12 ± 0.36∗	1.8 ± 1.54
*P*	/	0.216	0.583	0.0169/0.218	0.006

Note: ^∗^*P* < 0.05*vs* preoperative VAS score.

**Table 3:** Comparison of postoperative complication rates of patients with different surgical methods between the two groups (%).

Group	Cerebrospinal fluid leakage	Incision infection	Anemia	Residual nucleus pulposus	Incidence rate
UBE group (*n* = 22)	2 (9.1)	0 (0)	6 (27.3)	0 (0)	8 (36.4)
Interforaminal endoscopic group (*n* = 28)	0 (0)	0 (0)	0 (0)	7 (25.0)	7 (25.0)
*χ* ^2^	2.652	/	8.678	6.395	0.758
*P*	0.104	/	0.003	0.011	0.384

## Data Availability

The labeled dataset used to support the findings of this study are available from the corresponding author upon request.
